# Tube Feeding in Older Adults With Delirium: A Multicenter Study on Hospital Outcomes

**DOI:** 10.1093/geroni/igaf122.2005

**Published:** 2025-12-31

**Authors:** Marlon Juliano Romero Aliberti, Ezemir Dantas Fernandes Junior, Thiago Junqueira Avelino-Silva

**Affiliations:** University of Sao Paulo Medical School, Baltimore, Maryland, United States; University of Sao Paulo Medical School, Sao Paulo, Sao Paulo, Brazil; University of California, San Francisco, University of California, San Francisco, California, United States

## Abstract

Delirium is common in hospitalized older adults and is associated with malnutrition and dehydration. Enteral nutrition, including tube feeding, is often considered to ensure adequate intake, yet its impact on delirium and clinical outcomes remains debated. This study assessed the association between tube feeding and adverse hospital outcomes in hospitalized older adults with delirium. We conducted a prospective cohort study using data from the multicenter CHANGE (Creating a Hospital Assessment Network in Geriatrics) Study, which included patients aged ≥65 years diagnosed with delirium at hospital admission via the Confusion Assessment Method across 43 centers in five countries. Those receiving enteral or parenteral nutrition before hospitalization were excluded. The primary exposure was the initiation of tube feeding during hospitalization. Propensity score modeling and inverse probability of treatment weighting (IPTW) were applied to balance covariates. Among 566 participants (mean age=83±8 years; women=58%), 149 (26%) received tube feeding. Median hospital length of stay was 8 days (IQR=5-13) for those without tube feeding and 14 days (IQR=8-24) for those receiving tube feeding. Hospital mortality was 20% and 50%, respectively. Nosocomial pneumonia occurred in 9% of patients without and 35% with tube feeding. After IPTW adjustment, tube feeding was associated with higher in-hospital mortality (OR = 2.01; 95%CI=1.23-3.28) and nosocomial pneumonia (OR = 2.58; 95%CI=1.46-4.54), but not with length of hospital stay (OR = 0.99; 95%CI=0.94-1.04). In older adults with delirium, tube feeding was associated with higher in-hospital mortality and nosocomial pneumonia, without reducing length of stay. These findings warrant careful risk-benefit assessment when considering tube feeding in this population.

